# Response to: topical cyclosporine A 0.05% before and after surgery to prevent pterygium recurrence

**DOI:** 10.5935/0004-2749.20220092

**Published:** 2025-08-21

**Authors:** Goksu Alacamli

**Affiliations:** 1 Ophthalmology Departmant, Mugla University Education and Research Hospital, Mugla, Turkey

Dear Editor,

We have read with interest the article by Roberta Lilian Fernandes de Sousa Meneghim et al.^(1)^. In response to this article^(1)^ which is a well-thought out and written paper, I would like to draw attention to some critical points in this study. As presented in most studies, topical cyclosporine drops require at least 3-6 months for their effectiveness to begin^(2-4)^. In the article by Meneghim et al.^(1)^, topical cyclosporine was used for only 10 days before and after the operation. In our clinic, Mugla Education and Research Hospital, we prescribe topical cyclosporine 3 months before and 6 months after the pterygium operation. In this article^(1)^, it seemed obvious that topical cyclosporine used for such a short time before and after the operation would not have a statistical or clinical effect.

Pterygium pathogenesis has been mainly associated with ultraviolet light exposure; however, this association remains quite controversial. The complete pathophysiology of pterygium also remains to be clarified^(5)^. To reduce recurrences, new study and treatment methods are needed.
