# Supplementary material for: Response to: topical cyclosporine A 0.05% before and after surgery to prevent pterygium recurrence
Source: Arq Bras Oftalmol. 2025 Aug 21;85(2):202–4. doi: 10.5935/0004-2749.20220092 (PMC11826569; doi:10.5935/0004-2749.20220092)
Supplement: Related texts

# Reply: Response to: topical cyclosporine A 0.05% before and after surgery to prevent pterygium recurrence

**Authors:** Roberta Lilian Fernandes de Sousa Meneghim, Larissa Horikawa Satto, Kryscia Leiko Natsuaki, Alessandro Carvalho de Oliveira, Carlos Roberto Padovani, Magda Massae Hata Viveiros, Silvana Artioli Schellini

**Affiliations:** 1 Department of Ophthalmology, Botucatu Medical School, Universidade Estadual Paulista “Julio de Mesquita Filho”, Botucatu, SP, Brazil.; 2 Department of Biostatistic, Biosciences Institute, Universidade Estadual Paulista “Julio de Mesquita Filho”, Botucatu, SP, Brazil.

Dear Dr. Alacamli,

Thank you for your interest in our study involving pterygium and the use of cyclosporine A (CsA) as an adjuvant drug to prevent pterygium recurrence after lesion removal.

According to a recent review, the main risk factor of pterygium progression remains ultraviolet exposure^(1)^, and one of the most challenging aspects involving this lesion is still the prevention of recurrence.In a previous *in vitro* study, we found that 0.05% CsA is effective in inhibiting fibroblast proliferation, both in primary and in recurrent pterygium^(2)^. Also, *in vitro*, a combination of CsA with bevacizumab can reduce fibroblast outgrowth from cultured pterygium tissue explants, playing an important role in fibroblast migration and preventing T-helper cell activation and inflammatory cytokine production^(3)^.

However, when using CsA for 10 pre- and 10 postoperative days, as stated in our article, we observed that CsA did not prevent or reduce the recurrence of pterygium, probably because of the short period of use^(4)^. As you also reinforced, the results can be influenced by the short period that the drug was used for, which should be not enough.

In addition to the short period of use, there are other *biases* that can directly affect the outcome of the pterygium studies, as the inclusion of a few patients, application mode (topically or by subconjunctival injection), and variations in surgical techniques. Because of this, a meta-analysis was conducted, suggesting that adjuvant use of CsA can significantly reduce the risk of pterygium recurrence compared with pterygium excision alone, whereas CsA may not reduce the risk of pterygium recurrence if pterygium excision is associated with limbal conjunctival autograft or conjunctival flap rotation^(5)^.

Our study concluded that “Topical 0.05% CsA, when used for 10 days before and 10 days after the pterygium removal, does not prevent or reduce the recurrence of primary pterygium”, and we suggested that “further studies are necessary to evaluate the efficacy of CsA to prevent pterygium recurrence when used for different time periods and assess which number of days of preoperative CsA use provides benefit if any”.

According to others, postoperative topical 0.05% CsA (4 times a day for 6 months) can prevent recurrence of pterygium^(6)^.

In conclusion, further studies are still needed to prove the best way to prevent pterygium recurrence and the role of CsA in it.

## References

[1] Shahraki T, Arabi A, Feizi S (2021). Pterygium: an update on pathophysiology, clinical features,
and management. Ther Adv Ophthalmol..

[2] Viveiros MM, Kakizaki FY, Hercules LA, Padovani CR, Candeias JM, Schellini SA (2016). In vitro study of cyclosporine A 0.05% on primary and
recurrent pterygium fibroblasts. Int Ophthalmol.

[3] Kim YH, Jung JC, Gum S, Park SB, Ma JY, Kim Y (2017). Inhibition of pterygium fibroblast migration and outgrowth by
bevacizumab and cyclosporine A involves down-regulation of matrix
metalloproteinases- 3 and -13. PLoS One.

[4] Meneghim RLFS, Satto LH, Natsuaki KL, Oliveira AC, Padovani CR, Viveiros MMH (2019). Topical cyclosporine A 0.05% before and after surgery to
prevent pterygium recurrence. Arq Bras Oftalmol..

[5] Zhang Q, Bao N, Liang K, Tao L (2018). Adjuvant use of cyclosporine A in the treatment of primary
pterygium: a systematic review and meta-analysis. Cornea.

[6] Turan-Vural E, Torun-Acar B, Liang K, Kivanc SA, Acar S (2011). The effect of topical 0.05% cyclosporine on recurrence
following pterygium surgery. Clin Ophthalmol [Internet].
